# Preoperative Neutrophil-Lymphocyte Ratio and Platelet-Lymphocyte Ratio Are Not Clinically Useful in Predicting Prognosis in Early Stage Cervical Cancer

**DOI:** 10.1155/2018/9162921

**Published:** 2018-12-02

**Authors:** Prachratana Nuchpramool, Jitti Hanprasertpong

**Affiliations:** Division of Gynecologic Oncology, Department of Obstetrics and Gynecology, Faculty of Medicine, Prince of Songkla University, Songkhla 90110, Thailand

## Abstract

**Background:**

An objective of this study was to determine the prognostic role of neutrophil-lymphocyte ratio (NLR) and platelet-lymphocyte ratio (PLR) in patients with cervical cancer (CC) stages IA2-IB1.

**Methods:**

The study included 484 patients who underwent radical hysterectomy with pelvic node dissection. The associations of preoperative NLR and PLR with clinicopathologic characteristics and oncological outcomes were analyzed. The cut-off values of NLR (=1.8) and PLR (=119) were set as medians.

**Results:**

The clinicopathologic analysis showed that NLR was associated with age (*p*=0.010), tumor size (*p*=0.045), and adjuvant treatment (*p*=0.005), and PLR was associated with only adjuvant treatment (*p*=0.033). DFS and OS were not significantly different between patients with high and low NLR (*p*=0.670 and *p*=0.934) or high and low PLR (*p*=0.780 and *p*=0.306). The independent prognostic factors associated with OS were lymph node status and anemia, and with DFS were histology, deep stromal invasion, and lymph node status.

**Conclusions:**

NLR and PLR have no use as prognostic biomarker for DFS and OS in early-stage CC. However, NLR and PLR might be of use in determining the risk for adjuvant treatment.

## 1. Introduction

Although cervical cancer (CC) is considered to be one of the most preventable cancers, the clinical and economic burdens of this cancer are still meaningful issues in developing countries. The standard primary treatment of CC consists of radical hysterectomy with pelvic lymph node dissection (RHND), or radiotherapy (RT), or a combination of RT and platinum-based chemotherapy [1]. Generally agreed prognostic factors for CC include International Federation of Gynecology and Obstetrics (FIGO) stage, histological cell type, tumor size, parametrial involvement (PI), deep stromal invasion (DSI), and lymph node (LN) status [2–4]. However, we cannot use most of these prognostic factors (except, e.g., the FIGO stage) in preoperative prediction of estimated survival probability and prognosis in patients with early-stage CC. In addition, clinical staging in CC has been documented to be often inaccurate in predicting the prognosis of patients, especially in those with advanced stage [5, 6]. Consequently, identification of new prognostic markers that are accurate, reliable, and easy to use would be useful for stratification of patients into more accurate risk groups and provide more personalized medical treatment.

Over the past decade, there has been new evidence that cancer-related inflammation plays an important role in cancer development (such as cell proliferation, cell survival, and invasion) and its progression (such as metastasis) [7, 8]. Many systemic inflammatory markers such as serum C-reactive protein (CRP), neutrophil-lymphocyte ratio (NLR), or platelet-lymphocyte ratio (PLR) have been shown to be a prognostic marker in various kinds of human cancers such as lung, colorectal, ovarian, and endometrial cancer [9–12]. CRP is one of the well-established markers of systemic inflammation widely used in clinical practice [9]. However, CRP is not routinely measured as part of pretreatment evaluation of cancers. NLR and PLR have been suggested as simple and trusted markers of systemic inflammation, as they can be easily ascertained in cancer patients from a complete blood count.

Data from previous studies have shown that high-risk human papillomavirus infection (HPV), notably subtypes 16 and 18, is considered the most vital causative factor in carcinogenesis of CC, and inflammatory pathways play a vital role in tumorigenesis and progression [13, 14]. In 2012, a large retrospective study from Korea [15] reported that CC patients with a high NLR were younger in age and had more advanced disease when compared with those with low NLR. They also reported that pretreatment NLR was identified as an independent prognostic marker for poor oncological outcomes. Up to now, despite various prior studies having attempted to identify the prognostic role of NLR and PLR in CC, the results are conflicting [6,15–24]. Recently, one meta-analysis and systematic review based on data from 13 studies with 3,729 patients assessed the prognostic value of pretreatment NLR in CC and suggested that high pretreatment NLR predicted a poorer survival for CC patients [6]. However, the sample size of the majority of the included studies was small. Nearly 40% of all studies in this meta-analysis were retrospective studies without multivariate analyses. Moreover, this meta-analysis was limited to published papers, and data from studies with negative outcomes would unavoidably be missed. For early-stage CC, only scant and conflicting reports are available concerning the prognostic significance of NLR and PLR in patients with early-stage CC receiving initial RHND [15, 22–24].

We therefore evaluated the prognostic value of preoperative NLR and PLR in a large cohort of patients with early-stage CC treated with RHND. We also determined the association between preoperative NLR and PLR and the clinicopathologic characteristics of these patients.

## 2. Patients and Methods

This study was approved by the Ethics Committee of the Faculty of Medicine, Prince of Songkla University. A retrospective medical records review was performed in all patients with early-stage CC (stages IA2-IB1 by the FIGO 2009) who underwent an RHND (type II-III) at Songklanagarind Hospital from January 2001 to June 2016. Patients who had been diagnosed with other types of cancers (*n*=0), hematologic disease (*n*=0), acute urinary tract infection (*n*=1), or acute inflammatory disease (*n*=3) were excluded. This left 484 patients enrolled.

Clinicopathologic information of these patients, including age, stage, histology, tumor size, lymph vascular space invasion (LVSI), DSI, PI, LN status, vaginal involvement, adjuvant treatment, hemoglobin (Hb) level, white blood cell (WBC) count, platelet (PLT) count, NLR, PLR, and oncological outcomes, was collected.

Routine peripheral blood results were available as part of routine work-up and preoperative protocols. NLR was defined as the absolute neutrophil count divided by the absolute lymphocyte count. PLR was defined as the absolute PLT count divided by the absolute lymphocyte count. In our study, the cut-off values of preoperative NLR and PLR were set as the medians. Tumor-related anemia was defined as Hb < 11 g/dl without acute blood loss. Pretreatment WBC count of >10,000/*μ*l without known inflammatory condition or infectious disease was diagnostic for tumor-related leukocytosis. Thrombocytosis was defined as a pretreatment PLT count of >400,000/*μ*l without a known inflammatory condition [25].

At our hospital, whole pelvic radiation has been used as a postoperative treatment and is indicated when a patient's pathological report displays any of the following prognostic factors: PI, pelvic node metastasis, or positive surgical margin. However, since 2000, postoperative treatment protocols were based on pathological findings classified into 3 groups: low-, intermediate-, and high-risk groups, these being based on eligibility for Gynecologic Oncology Group (GOG) 92 [26] and GOG 109 [27]. Patients with any one of the factors such as PI, pelvic node metastasis, or positive surgical margin were classified into the high-risk group. Patients with 2 or more of the factors such as tumor size >4 cm, LVSI, or DSI were classified into the intermediate-risk group. Patients within the high-risk group were recommended to receive postoperative concurrent chemoradiation with cisplatin. However, in the high-risk group, patients who either had poor performance status or refused chemotherapy received pelvic radiation therapy alone. Patients within the intermediate-risk group were recommended to undergo postoperative radiation therapy alone. Patients without any factors were considered to be in the low-risk group and did not receive adjuvant treatment [4].

After complete treatment, the patients had follow-up examinations in the outpatient clinic at approximately every 3 months for the 1st year, every 4 months for the 2nd year, every 6 months for the 3rd to 5th years, and annually thereafter. If recurrence occurred, the time to recurrence would be recorded. The primary outcome measures were disease-free survival (DFS) and overall survival (OS) [1].

Descriptive statistics of patient characteristics were analyzed using frequency and percentage. Low- and high-ratio (NLR or PLR) groups were compared using the chi-square test for categorical or ordinal variables and the log-rank test for survival data. Associations between potential risk factors and the occurrence of low NLR or PLR were identified using tabulation and univariate logistic regression models, followed by multiple logistic regression models. The significance of each variable in the models was evaluated using the likelihood ratio test. All analyses were conducted using STATA version 14 (Stata Corporation, College Station, TX, USA). A *p* value less than 0.05 was considered statistically significant. No adjustment was made for multiple testing.

## 3. Results

A total of 484 patients were analyzed. The median age was 47 years (25% quartile = 40 years, 75% quartile = 54 years). The median follow-up time was 56.9 months (25% quartile = 26.1 months, 75% quartile = 102.9 months). Overall, 15 patients died, and 40 had had recurrence at the time of analysis. The 5-year DFS and 5-year OS for the entire population were 88.3% (95% confidence interval (CI) = 84.5–91.2) and 96.9% (95% CI = 94.2–98.4), respectively.

Among the 484 patients, data on both NLR and PLR were missing from 22, and data on PLR alone were missing from 2 patients. Analysis to identify predictors of NLR and PLR was confined to those patients without missing outcomes. However, survival analysis was based on all 484 patients.

In this study, the median NLR and PLR were 1.8 and 119, respectively. The clinicopathological characteristics of early-stage CC patients according to NLR are shown in Table 1. Patients with a high NLR (>1.8) were more likely to have older age (*p*=0.010) and tumor size more than 2 cm (*p*=0.045) and received adjuvant treatment (*p*=0.005). However, the NLR was not significantly associated with stage, histology, LVSI, DSI, PI, LN status, or vaginal margin.  Table 2 shows the clinicopathological characteristics of early-stage CC patients according to PLR. As seen in this table, patients with a high PLR were more likely to have received adjuvant treatment (*p*=0.033). There were no significant differences in age, stage, histology, tumor size, LVSI, DSI, PI, LN status, or vaginal margin between the 2 groups (high PLR and low PLR).

**Figure 1:**
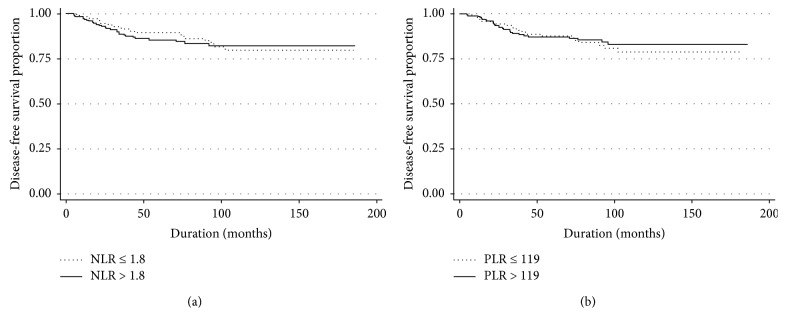
The disease-free survival according to neutrophil/lymphocyte ratio (a) and platelet/lymphocyte ratio (b).

**Figure 2:**
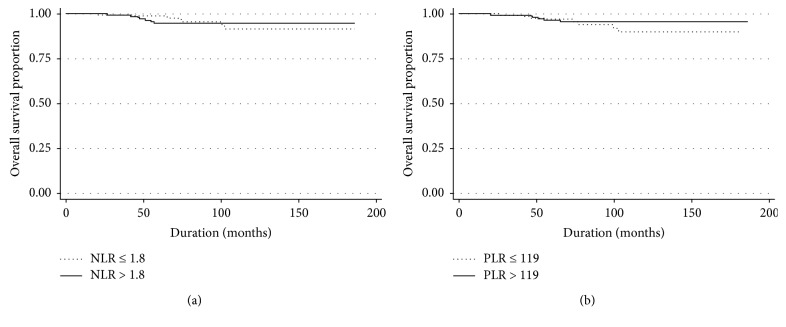
The overall survival according to neutrophil/lymphocyte ratio (a) and platelet/lymphocyte ratio (b).

Table 3 provides the results of univariate analysis of DFS and OS in early-stage CC patients. DFS did not differ significantly with NLR (≤1.8 vs. > 1.8, *p*=0.670) (Figure 1(a)) and PLR (≤119 vs. > 119, *p*=0.078) (Figure 1(b)). OS also did not differ with NLR (≤1.8 vs. > 1.8, *p*=0.934) (Figure 2(a)) and PLR (≤119 vs. > 119, *p*=0.306) (Figure 2(b)). Age (*p*=0.024), histology (*p* < 0.001), LVSI (*p*=0.027), DSI (*p* < 0.001), PI (*p*=0.006), LN status (*p* < 0.001), vaginal margin (*p*=0.007), adjuvant treatment (*p*=0.024), and Hb level (*p*=0.009) were associated with DFS, while PI (*p*=0.002), LN status (*p* < 0.001), and Hb level (*p*=0.016) were associated with OS. Further multivariate analysis showed that histology (HR = 3.4, 95% CI = 1.5–7.8 for adenosquamous carcinoma and HR = 6.9, 95% CI = 2.8–17.1 for other cell type; *p*=0.001), DSI (HR = 2.5, 95% CI = 1.5–4.4; *p*=0.001), and LN status (HR = 3.0, 95% CI = 1.4–6.4; *p*=0.013) were identified as independent poor prognostic factors for DFS (Table 4). The statistically significant independent poor prognostic factor for OS were LN status (HR = 9.6, 95% CI = 2.9–31.4; *p*=0.002) and Hb level (HR = 4.5, 95% CI = 1.3–14.7; *p*=0.027) (Table 5).

## 4. Discussion

As mentioned in the literature review, various associations between pretreatment NLR and PLR and the clinicopathologic characteristics of CC patients, including age of patient, FIGO stage, tumor size, tumor differentiation, LVSI, DSI, and LN status, have been reported [6, 15, 17, 20, 23, 24]. In our study of 484 patients with early-stage CC, we found that a high NLR was more likely to be found in older patients and patients with a larger tumor size. This finding, which is in line with previous studies [17, 18], reported that cervical cancer patients with a high NLR were more likely to have large tumor size. However, we found that there were no significant differences between patients with either high NLR or low NLR in stage, histology, LVSI, DSI, PI, LN status, or vaginal margin. We also found that a high PLR was not associated with many clinicopathologic characteristics including age of patient, stage, histology, tumor size, LVSI, DSI, PI, LN status, or vaginal margin. These differences between ours and other studies can be explained in part by different patient characteristics and/or sample sizes. Surprisingly, we found that patients with a high NLR and a high PLR were more likely to have received adjuvant treatment. Thus, these findings highlight the potential clinical value of both pretreatment NLR and PLR for determining the risk for adjuvant treatment after RHND. To our knowledge, our study is the first to show these associations, and further future investigations are needed to explore the possible significance of these findings.

Regarding the association between the pretreatment NLR and PLR and oncological outcomes, our study found that the pretreatment NLR and PLR were not associated with poor oncological outcomes (DFS and OS) in patients with early-stage CC treated with RHND. In the last 4 years, there has been growing interest in the possible prognostic value of the pretreatment NLR and PLR in CC. In 2014, Zhang et al. [22] studied the prognostic values of NLR and PLR in 460 patients with CC stages I-II treated with initial RHND and found that the pretreatment NLR, but not PLR, can be used as a potential marker to help determine survival prognosis in these patients. Furthermore, a study by Mizunuma et al. [18], which assessed 56 patients with squamous cell CC stages I-IV, found that the high pretreatment NLR was associated with poor progressive-free survival and OS. More recently, Cheng et al. [20] found that NLR was an independent prognostic marker for DFS and OS in CC. However, these findings are in disagreement with the findings of some former studies [16, 23, 24] and our study, which found that NLR was not associated with poor clinical outcomes. Wang et al. [23] investigated the predictive values of the pretreatment NLR, PLR, and red cell distribution width in 515 patients with squamous cell CC stages I-IV and found that NLR and PLR might be able to predict LN and distant metastasis in these patients, but were not adequate prognostic indicators for early-stage CC.

The high predictive value of PLR has been demonstrated to be a negative prognostic factor for many human cancers including CC [12, 19, 20, 24, 28], although some studies have reported negative results as well, finding that the pretreatment PLR is not to be associated with the oncological outcomes of CC patients [16, 21, 23]. This study is in agreement with the latter studies, as no prognostic value of preoperative PLR was found in our patients with CC stages IA2-IB1 treated with RHND. These contrasting results may partly be explained by weaker correlations that are not uniformly present in the different studies because of small sample size or heterogeneity within study populations (such as tumor stage and histology) or short duration of follow-up time or variation in study design. For example, all the patients in our study and one study [24] with negative results had early-stage disease with relatively small tumor size in which prognosis is generally quite good after treatment, while most of the studies [6, 15, 19] with positive results had patients with more advanced stage disease, in which recurrence and mortality rates are high. Furthermore, the number of patients in this study and some studies [21, 23] with similar results may not have been enough to detect the prognostic value of pretreatment NLR and PLR in early-stage CC. Another possible explanation for these contrasting results is the varying of the pretreatment NLR and PLR cut-off values used in each study because the cut-off values of the pretreatment NLR and PLR in each study were obtained using different methods.

The specific biological mechanisms involved in as to the association between the high NLR and PLR and poor prognosis for CC patients remain unclear. Changes in NLR and PLR indicate the balance between host neutrophil- and PLT-dependent inflammatory responses and lymphocyte-mediated antitumor immune responses [23]. Neutrophils have been shown to contain and release most of the circulating vascular endothelial growth factors (VEGF) that are thought to be involved in cancer development [29]. Consequently, an elevated neutrophil level stimulates tumor angiogenesis and assists cancer progression. Patients with a high NLR have relative lymphopenia, which may indirectly suggest an imperfect lymphocyte-mediated immune response to cancer [12]. Circulating lymphocytes, such as CD3^+^ T cells, CD8^+^ T cells, and natural killer cells, play an important role in preventing the proliferation and metastasis of cancer cells [30]. Cytokines such as VEGF and transforming growth factor β are meaningful in tumor angiogenesis. PLTs are considered the major sources of those cytokines. In some situations, PLT levels become higher than normal, for example, when cancers or inflammatory cells release inflammatory mediators that can stimulate megakaryocyte-release PLTs [31]. One study has suggested that PLTs may participate in cancer progression and metastasis [30].

Our study has some limitations that have to be pointed out. First, this was a retrospective study from a single center based on a relatively small number of records of patients with early-stage CC, and thus it was difficult to control for potential confounding factors that may affect preoperative NLR and PLR. Second, the relatively small number of patients prevented us from statistically significant analysis of the survivor factors. Consequently, further large-scale studies with a long period of follow-up with standardized investigations are needed to confirm our findings. The optimal cut-off values of NLR and PLR could be standardized in future studies too.

In conclusion, although assessing preoperative NLR and PLR levels is simple and inexpensive based on the available complete blood counts, our study indicates that they do not have a role as prognostic biomarkers for DFS and OS in early-stage CC after RHND. However, they might be useful in determining the risk for adjuvant treatment.

## Figures and Tables

**Table 1:** Clinicopathological characteristics according to neutrophil/lymphocyte ratio.

Variable	*n* (%) (*n*=462)	Neutrophil/lymphocyte ratio		*p* value
		≤1.8 (*n*=242)	>1.8 (*n*=220)	
Age				0.011
** **<50 years	162 (35.1)	98 (40.5)	64 (29.1)	
** **≥50 years	300 (64.9)	144 (59.5)	156 (70.9)	
Stage				0.195
** **IA2	42 (9.1)	26 (10.7)	16 (7.3)	
** **IB1	420 (90.9)	216 (89.3)	204 (92.7)	
Histology				0.424
** **Squamous carcinoma	270 (58.5)	134 (55.4)	136 (61.8)	
** **Adenocarcinoma	153 (33.1)	86 (35.5)	67 (30.5)	
** **Adenosquamous carcinoma	26 (5.6)	16 (6.6)	10 (4.5)	
** **Others	13 (2.8)	6 (2.5)	7 (3.2)	
Tumor size				0.045
** **≤2 cm	311 (67.3)	173 (71.5)	138 (62.7)	
** **>2 cm	151 (32.7)	69 (28.5)	82 (37.3)	
Lymph vascular space invasion				0.630
** **No	347 (75.1)	184 (76.0)	163 (74.1)	
** **Yes	115 (24.9)	58 (24.0)	57 (25.9)	
Deep stromal invasion				0.353
** **No	341 (73.8)	183 (75.6)	158 (71.8)	
** **Yes	121 (26.2)	59 (24.4)	62 (28.2)	
Parametrial involvement				0.982
** **No	443 (95.9)	232 (95.9)	211 (95.9)	
** **Yes	19 (4.1)	10 (4.1)	9 (4.1)	
Lymph node status				0.280
** **No	438 (94.8)	232 (95.8)	206 (93.6)	
** **Yes	24 (5.2)	10 (4.1)	14 (6.4)	
Vaginal margin				0.837
** **No	444 (96.1)	233 (96.3)	211 (95.9)	
** **Yes	18 (3.9)	9 (3.7)	9 (4.1)	
Adjuvant treatment				0.005
** **No	368 (79.7)	205 (84.7)	163 (74.1)	
** **Yes	94 (20.3)	37 (15.3)	57 (25.9)	

**Table 2:** Clinicopathological characteristics according to platelet/lymphocyte ratio.

Variable	*n* (%) (*n*=460)	Platelet/lymphocyte ratio		*p* value
		≤119 (*n*=231)	>119 (*n*=229)	
Age				0.270
** **<50 years	162 (35.2)	87 (37.7)	75 (32.8)	
** **≥50 years	298 (64.8)	144 (62.3)	154 (67.2)	
Stage				0.537
** **IA2	42 (9.1)	23 (10.0)	19 (8.3)	
** **IB1	418 (90.9)	208 (90.0)	210 (91.7)	
Histology				0.711
** **Squamous carcinoma	268 (58.3)	129 (55.9)	139 (60.7)	
** **Adenocarcinoma	153 (33.2)	80 (34.6)	73 (31.9)	
** **Adenosquamous carcinoma	26 (5.7)	15 (6.5)	11 (4.8)	
** **Others	13 (2.8)	7 (3.0)	6 (2.6)	
Tumor size				0.574
** **≤2 cm	309 (67.2)	158 (68.4)	151 (65.9)	
** **>2 cm	151 (32.8)	73 (31.6)	78 (34.1)	
Lymph vascular space invasion				0.627
** **No	346 (75.2)	176 (76.2)	170 (74.2)	
** **Yes	114 (24.8)	55 (23.8)	59 (25.8)	
Deep stromal invasion				0.222
** **No	339 (73.7)	176 (76.2)	163 (71.2)	
** **Yes	121 (26.3)	55 (23.8)	66 (28.8)	
Parametrial involvement				0.470
** **No	441 (95.9)	223 (96.5)	218 (95.2)	
** **Yes	19 (4.1)	8 (3.5)	11 (4.8)	
Lymph node status				0.691
** **No	436 (94.8)	218 (94.4)	218 (95.2)	
** **Yes	24 (5.2)	13 (5.6)	11 (4.8)	
Vaginal margin				0.617
** **No	442 (96.1)	223 (96.5)	219 (95.6)	
** **Yes	18 (3.9)	8 (3.5)	10 (4.4)	
Adjuvant treatment				0.033
** **No	366 (79.6)	193 (83.5)	173 (75.5)	
** **Yes	94 (20.4)	38 (16.5)	56 (24.5)	

**Table 3:** Univariate analysis of disease-free survival and overall survival.

Variable	*n* (%) (*n*=484)	Disease-free survival		Overall survival	
		5-year DFS% (95% CI)	*p* value^*∗∗*^	5-year OS% (95% CI)	*p* value^*∗∗*^
Age			0.024		0.448
** **<50 years	170 (35.1)	86.6 (79.1–91.5)		97.9 (91.6–99.5)	
** **≥50 years	314 (64.9)	89.2 (84.6–92.5)		96.5 (92.8–98.3)	
Stage			0.066		0.238
** **IA2	45 (9.3)	97.1 (81.4–99.6)		100	
** **IB1	439 (90.7)	87.5 (83.3–90.6)		96.7 (93.7–98.3)	
Histology			<0.001		0.644
** **Squamous carcinoma	285 (58.9)	91.1 (86.5–94.2)		96.6 (92.6–98.5)	
** **Adenocarcinoma	158 (32.6)	90.1 (83.2–94.3)		98.8 (91.7–99.8)	
** **Adenosquamous carcinoma	28 (5.8)	63.8 (36.7–81.7)		95.0 (69.5–99.3)	
** **Others	13 (2.7)	56.3 (24.4–79.1)		85.7 (33.4–97.9)	
Tumor size			0.072		0.125
** **≤2 cm	326 (67.4)	91.1 (86.9–94.0)		98.4 (95.2–99.5)	
** **>2 cm	158 (32.6)	82.2 (73.8–88.1)		93.5 (85.9–97.1)	
Lymph vascular space invasion			0.027		0.404
** **No	360 (74.4)	90.5 (86.3–93.4)		97.6 (94.4–99.0)	
** **Yes	124 (25.6)	82.1 (72.7–88.6)		94.9 (86.9–98.1)	
Deep stromal invasion			<0.001		0.181
** **No	359 (74.2)	92.2 (88.4–94.8)		97.6 (94.4–99.0)	
** **Yes	125 (25.8)	76.1 (65.7–83.7)		94.8 (86.6–98.1)	
Parametrium involvement			0.006		0.002
** **No	465 (96.1)	89.4 (85.7–92.2)		97.7 (95.0–99.0)	
** **Yes	19 (3.9)	64.2 (36.9–82.1)		80.8 (51.4–93.4)	
Lymph node status			<0.001		<0.001
** **No	460 (95.0)	89.7 (86.0–92.4)		98.1 (95.6–99.2)	
** **Yes	24 (5.0)	61.8 (36.0–79.7)		71.3 (39.2–88.5)	
Vaginal margin			0.007		0.534
** **No	466 (96.3)	89.1 (85.3–92.0)		97.1 (94.3–98.6)	
** **Yes	18 (3.7)	70.6 (43.2–86.6)		93.8 (63.2–99.1)	
Adjuvant treatment			0.024		0.201
** **No	385 (79.6)	90.5 (86.5–93.3)		98.2 (95.2–99.3)	
** **Yes	99 (20.4)	80.7 (69.9–88.0)		98.2 (95.2–99.3)	
Hemoglobin^*∗*^			0.009		0.016
** **≥11 g/dl	430 (89.6)	90.0 (86.2–92.8)		98.0 (95.2–99.2)	
** **<11 g/dl	50 (10.4)	73.8 (57.3–84.8)		86.8 (67.8–95.0)	
White blood cell^*∗*^			0.468		0.892
** **≤10,000/*µ*l	409 (85.4)	87.8 (83.7–91.0)		97.2 (94.2–98.7)	
** **>10,000/*µ*l	70 (14.6)	90.5 (78.6–96.0)		95.3 (82.4–98.8)	
Platelet^*∗*^			0.473		0.926
** **≤400,000/*µ*l	454 (94.8)	88.1 (84.2–91.1)		97.1 (94.2–98.6)	
** **>400,000/*µ*l	25 (5.2)	90.2 (66.2–97.5)		94.1 (65.0–99.2)	
NLR^*∗*^			0.670		0.934
** **≤1.8	242 (52.4)	89.7 (84.2–93.3)		98.7 (94.9–99.7)	
** **>1.8	220 (47.6)	85.6 (79.3–90.1)		94.8 (89.4–97.5)	
PLR^*∗*^			0.780		0.306
** **≤119	231 (50.2)	87.9 (81.7–92.1)		96.8 (91.5–98.8)	
** **>119	229 (49.8)	87.3 (81.6–91.3)		96.7 (92.2–98.6)	

^*∗*^Numbers may not sum to total because of missing data, ^*∗∗*^*p* value based on nonmissing data. NLR = neutrophil/lymphocyte ratio; PLR = platelet/lymphocyte ratio; DFS = disease-free survival; OS = overall survival; CI = confidence interval.

**Table 4:** Multivariate analysis of disease-free survival.

Variable	Hazard ratio	95% confidence interval	*p* value
Histology			0.001
** **Squamous carcinoma	1	—	
** **Adenocarcinoma	1.5	0.8–2.8	
** **Adenosquamous carcinoma	3.4	1.5–7.8	
** **Others	6.9	2.8–17.1	
Deep stromal invasion			0.001
** **No	1	—	
** **Yes	2.5	1.5–4.4	
Lymph node status			0.013
** **No	1	—	
** **Yes	3.0	1.4–6.4	
NLR			0.658
** **≤1.8	1	—	
** **>1.8	1.1	0.6–2.1	
PLR			0.808
** **≤119	1	—	
** **>119	0.9	0.5–1.7	

NLR = neutrophil/lymphocyte ratio; PLR = platelet/lymphocyte ratio.

**Table 5:** Multivariate analysis of overall survival.

Variable	Hazard ratio	95% confidence interval	*p* value
Lymph node status			0.002
** **No	1	—	
** **Yes	9.6	2.9–31.4	
Hemoglobin			0.027
** **≥11 g/dl	1	—	
** **<11 g/dl	4.5	1.3–14.7	
NLR			0.900
** **≤1.8	1	—	
** **>1.8	1.1	0.3–3.6	
PLR			0.232
** **≤119	1	—	
** **>119	0.5	0.1–1.6	

NLR = neutrophil/lymphocyte ratio; PLR = platelet/lymphocyte ratio.

## Data Availability

The data used to support the findings of this study are available from the corresponding author upon request.
